# Eagle’s syndrome with stylohyoid chain pseudoarthrosis and thyrohyoid ligament ossification: A case report and literature review

**DOI:** 10.1097/MD.0000000000045303

**Published:** 2025-10-17

**Authors:** Hui Li, Si-Qing Zhang, Lei Zhang, He Liu, Hai-Ying Zheng

**Affiliations:** a Department of Stomatology, Affiliated Hospital of Jining Medical University, Jining, China; b Department of Oral Biological and Medical Sciences, Faculty of Dentistry, University of British Columbia, Vancouver, Canada.

**Keywords:** case report, Eagle’s syndrome, stylohyoid chain, stylohyoid ligament ossification, thyrohyoid ligament

## Abstract

**Rationale:**

Eagle’s syndrome is caused by anatomical variations or ossification of the stylohyoid chain (SHC) and presents with complex, nonspecific symptoms, often leading to delayed diagnosis. Complete ossification of the SHC is exceptionally rare, and reporting such cases may enhance clinical awareness and improve diagnostic accuracy.

**Patient concerns:**

A 57-year-old man presented with persistent right mandibular pain lasting over 4 months. The pain intensified with mouth opening, swallowing, or head tilting, and radiated beneath the right earlobe. Conservative treatments including anti-inflammatory medication, nerve block therapy, and acupuncture were ineffective.

**Diagnoses:**

Physical examination revealed tenderness and swelling under the right earlobe, pain in the right floor of the mouth, and moderate limitation of mouth opening. Multilayer spiral CT with 3-dimensional reconstruction demonstrated complete ossification and thickening of the right SHC, elongation of the left styloid process, and ossification of the left lateral thyrohyoid ligament, confirming Eagle’s syndrome.

**Interventions:**

Extraoral surgical resection was performed, including excision of the ossified stylohyoid ligament, the entire styloid process, and partial hyoid bone resection.

**Outcomes:**

The patient achieved complete pain relief postoperatively. During a long-term follow-up of nearly 5 years, no recurrence was observed.

**Lessons:**

This case illustrates an uncommon presentation of Eagle’s syndrome with complete SHC ossification and thyrohyoid ligament involvement. Multilayer spiral CT with 3-dimensional reconstruction provides critical diagnostic and surgical planning value. Surgical resection remains the most effective treatment and should be individualized according to the patient’s anatomical features and symptoms.

## 1. Introduction

The stylohyoid chain (SHC), also known as the stylohyoid complex, comprises the styloid process (SP), stylohyoid ligament (SHL), and the lesser horn of the hyoid bone, forming a bone-ligament complex that connects the skull to the hyoid bone.^[1]^ The SHC is anatomically complex and displays a wide range of variations, including changes in the length of the SP (absence, duplication, and elongation), varying degrees of ossification of the SHC (segmental and complete), and fusions within parts of the SHC (fractures and pseudoarthrosis).^[2–4]^ While elongation of the SP or partial ossification of the SHL is not uncommon,^[5–9]^ complete ossification of the entire SHC is extremely rare.^[10,11]^ A forensic autopsy report of 1215 cases indicated an occurrence rate of complete ossification of the SHC at only 0.09%.^[11]^

Anatomical variations in the SHC that compress surrounding neural and vascular structures can result in Eagle’s syndrome, first described by Watt Eagle in 1937.^[5,6,12]^ This condition is characterized by a spectrum of symptoms such as vague myofascial, oropharyngeal, craniofacial, or cervical pain, temporomandibular joint disorders, tinnitus, excessive salivation, foreign body sensation in the throat, dysphagia, voice changes, and recurrent syncope.^[13–17]^ The clinical presentation is highly variable, and patients may exhibit one or several symptoms that can fluctuate over time, making accurate diagnosis particularly challenging.^[13–17]^

Several cases of Eagle’s syndrome have been reported in the literature, underscoring its diverse clinical manifestations and the importance of individualized management.^[18–20]^ For example, Aldelaimi et al described a 30-year-old male who presented with facial pain and restricted mouth opening due to an elongated SP.^[18]^ The diagnosis was confirmed radiographically, and the patient underwent intraoral styloidectomy with resolution of symptoms at follow-up. Such reports highlight the therapeutic options available, which range from conservative measures, including analgesics, anti-inflammatory agents, and steroid infiltrations, to definitive surgical resection via intraoral or extraoral approaches. The intraoral route offers the advantage of avoiding cutaneous scarring but provides limited surgical access and carries a risk of incomplete resection or deep cervical infection. The extraoral approach allows for better visualization and safer resection of ossified segments but is associated with longer recovery, scarring, and a risk of facial nerve injury. These reports emphasize the need for comprehensive evaluation, advanced imaging, and tailored surgical planning in managing Eagle’s syndrome.

This article presents a unique case of Eagle’s syndrome with complete ossification of the SHC, featuring a surgically excised bone fragment measuring 10 cm, inclusive of a pseudoarthrosis. The ossified tissue extended approximately 6.5 cm from the pseudoarthrosis to the skull and 3.5 cm from the pseudoarthrosis to the hyoid bone. In addition, the patient exhibited an elongated left SP and ossification of the left lateral thyrohyoid ligament. Despite these abnormalities, the patient experienced no symptoms on the left side and was placed under observation. To the best of our knowledge, this is the first reported case in the medical literature describing such extensive ossification of the SHC in conjunction with ossification of the lateral thyrohyoid ligament.

## 2. Case report

A 57-year-old male Chinese patient was admitted to the Department of Stomatology, Affiliated Hospital of Jining Medical University with the chief complaint of persistent discomfort and pain in the right lower jaw for over 4 months. The patient reported that the pain, which began without any apparent cause, initially occurred only during jaw movement and was not addressed at the time. One month prior to admission, the pain intensified during mouth opening, swallowing, and head tilting, accompanied by pain under the right earlobe, significantly affecting his eating habits. The patient had previously taken oral antibiotics without relief and was diagnosed with “jawbone inflammation” at a local hospital, where he underwent “nerve block therapy” once a week for a month, but the pain persisted. A week before hospitalization, he sought treatment at a local traditional Chinese medicine hospital and underwent acupuncture daily for 7 days, again without pain relief. The patient denied any history of surgery or trauma, reported allergies to prednisone and neomycin, and had no harmful habits.

**Figure 1.**
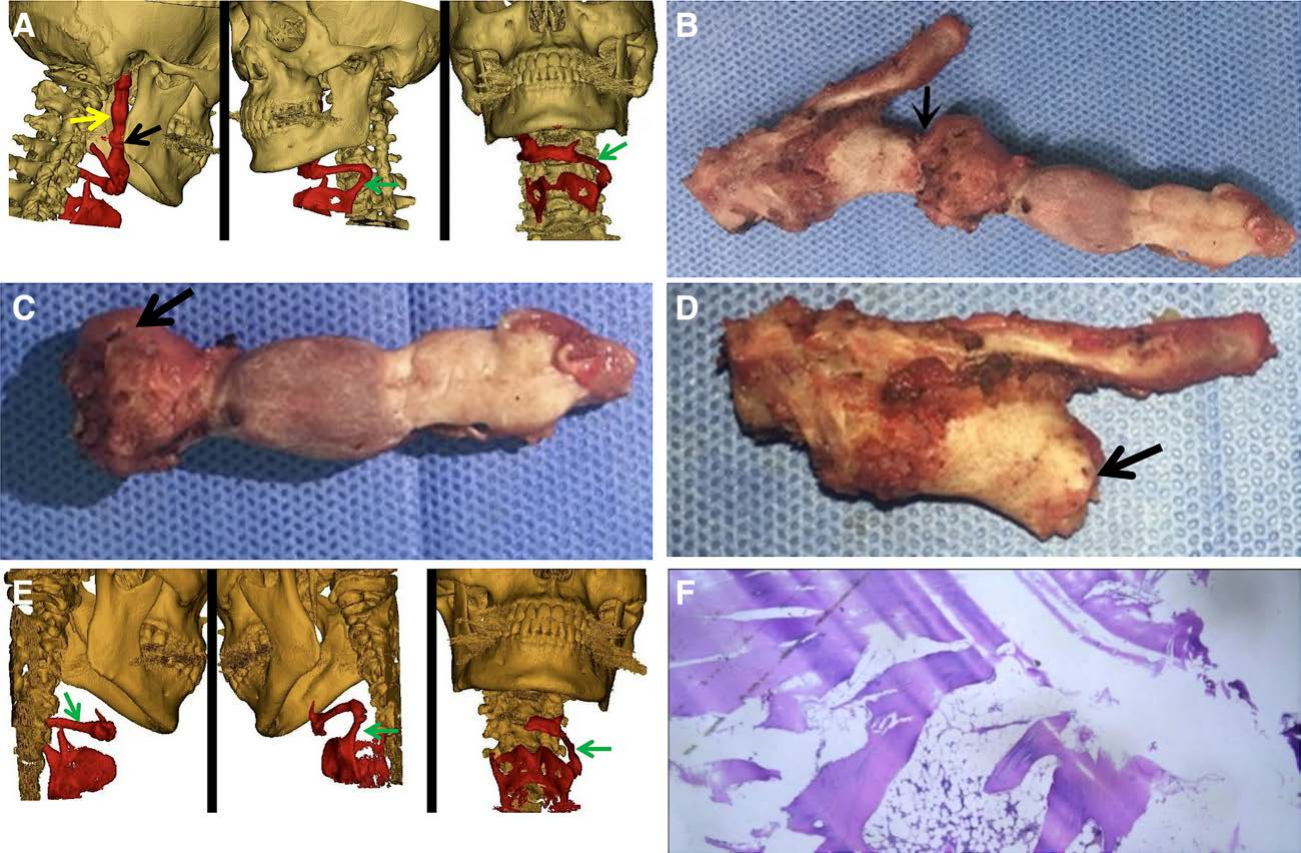
(A) The preoperative reconstructed image was formed after processing with Mimics 20.0 software (Materialise, Leuven, Belgium), following a multilayer spiral CT scan with 3D reconstruction. The area marked in red shows a completely ossified stylohyoid chain (yellow arrow) on the right side with pseudoarthrosis formation (black arrow). Additionally, there is ossification and thickening at the junction of the stylohyoid ligament and the lesser horn of the hyoid bone, and complete ossification of the left lateral thyrohyoid ligament (green arrow). (B) The right stylohyoid chain, approximately 10 cm in length, was excised during surgery and sent for histopathological examination. The position of the pseudoarthrosis is indicated by the black arrow. (C) The ossified tissue between the pseudoarthrosis (black arrow) and the skull measures approximately 6.5 cm in length. (D) The ossified tissue between the pseudoarthrosis (black arrow) and the hyoid bone is approximately 3.5 cm in length. (E) The postoperative reconstructed image was formed after processing with Mimics 20.0 software, following a multilayer spiral CT scan with 3D reconstruction. The area marked in red shows a partial defect in the hyoid bone and complete ossification of the left lateral thyrohyoid ligament (green arrow). (F) Histopathological section of the ossified stylohyoid chain showing mature trabecular bone formation and bone marrow-like adipose tissue (hematoxylin and eosin staining, original magnification ×40). The presence of lamellar bone and marrow spaces confirms extensive ossification, consistent with the diagnosis of Eagle’s syndrome caused by complete ossification of the stylohyoid chain. 3D = three-dimensional, CT = computed tomography.

The patient reported no significant family history of related illnesses. The patient’s vital signs were stable, with a body temperature of 36.3°C, a pulse rate of 77 beats/min, a respiratory rate of 19 breaths/min, and a blood pressure of 128/80 mm Hg. Clinical examination revealed that the patient had a limited mouth opening of <2 fingers width, indicating moderate trismus. Intraorally, tooth #46 was missing, and there were no protrusions in the oral floor mucosa, but palpation of the right side of the oral floor elicited pain. Tenderness was noted under the right earlobe, and a palpable swelling was detected under the right jaw, measuring approximately 2 cm × 2 cm. The swelling was hard, poorly mobile, with unclear boundaries, and tender upon palpation. The patient underwent routine preoperative examinations, including blood tests, electrocardiogram, and chest x-ray, which showed no significant abnormalities. The multilayer spiral CT with 3-dimensional reconstruction revealed complete ossification of the right SHC, with the widest part measuring approximately 1.0 cm × 1.3 cm; the left SP was about 4.4 cm long; and there was ossification of the left lateral thyrohyoid ligament (Fig. 1A).

Based on these findings, the diagnosis of Eagle’s syndrome with complete ossification of the right SHC was thus confirmed. Prognostic characteristics were complete ossification with pseudoarthrosis, no trauma history, and suitability for extraoral resection, suggesting favorable outcome. As conservative interventions, including antibiotics, nerve block therapy, and acupuncture, proved ineffective, the management strategy was shifted to surgical resection. Despite bilateral abnormalities, intervention was restricted to the symptomatic right side to minimize unnecessary surgical risk. Following a detailed discussion of treatment options, the patient consented to undergo extraoral resection.

The surgery was performed under general anesthesia with the patient in a supine position, shoulders elevated, head turned to the left side, and the lower jaw raised. A 6 cm incision was made in the neck, extending from below the chin to behind the ear, cutting through the skin, subcutaneous tissue, and platysma to expose the submandibular triangle. Blunt dissection was performed along the lower edge of the submandibular gland capsule to expose the intermediate tendon of the digastric muscle, which was then cut and ligated along with the external maxillary artery and lingual artery. Further blunt dissection exposed the right greater horn of the hyoid bone, which appeared abnormally calcified and irregular, along with the ossified SHL above it. Continuing the dissection anterior to the sternocleidomastoid muscle, the upper ossified SHL was fully exposed. A specimen approximately 10 cm in length, including the ossified SHL, SP, and part of the hyoid bone, was excised in its entirety (Fig. 1B). The area was irrigated, sutured, and a negative pressure drainage tube was placed with pressure bandaging. It was crucial to remove the ossified tissue as close to the skull base as possible to prevent re-ossification at the distal end. During surgery, the ossified SHC was found to be divided by a pseudoarthrosis into 2 segments of different lengths (Fig. 1C, D). No adverse or unanticipated events occurred intraoperatively.

Postoperatively, the patient received anti-infection treatment for 5 days. The negative pressure drainage tube was removed on the third day after surgery. The sutures were removed on the seventh day after surgery, and the patient was discharged.

On the sixth day post-surgery, a follow-up multilayer spiral CT with 3D reconstruction was performed (Fig. 1E). The results showed an irregular mass-like soft tissue density in the right surgical area and partial bone loss on the right side. Pathological analysis revealed mature dense bone tissue, confirming the diagnosis of ossification of the SHC (Fig. 1F). At a follow-up of over 4 years and eleven months, the patient’s symptoms had completely resolved, with no recurrence.

The timeline of key milestones in diagnosis and interventions is summarized in Table 1.

**Table 1:** Timeline of key milestones in diagnosis and interventions.

Time point	Clinical events/interventions		Findings/outcome
>4 mo before admission		Onset of right mandibular pain without obvious cause, aggravated by jaw movement	Pain not treated initially
1 mo before admission		Pain intensified with mouth opening, swallowing, head tilting; radiated under right earlobe	Eating habits affected
Local hospital visit		Oral antibiotics prescribed; diagnosed with “jawbone inflammation”	No relief
Weekly for 1 mo		Nerve block therapy	Ineffective
1 wk before admission		Acupuncture at local traditional Chinese medicine hospital for 7 d	No relief
Admission (day 0)		To Department of Stomatology, Affiliated Hospital of Jining Medical University	Persistent right mandibular pain
Initial examination		Stable vital signs; moderate trismus (<2 fingers); swelling (2 cm × 2 cm) under right jaw; tenderness under earlobe; missing tooth #46	Suggestive of pathology
Imaging		Multilayer spiral CT with 3D reconstruction	Complete ossification of right stylohyoid chain; elongated left styloid process; ossified left lateral thyrohyoid ligament
Diagnosis		Eagle’s syndrome with complete ossification of right stylohyoid chain	Confirmed
Surgery (extraoral approach)		6 cm neck incision; dissection; removal of ossified SHC + styloid process + part of hyoid bone (~10 cm specimen)	Complete excision achieved
Early postoperative course		5 d antibiotics; drainage tube removed day 3; sutures removed day 7	Stable recovery
Postoperative day 6 imaging		CT with 3D reconstruction	Soft tissue changes; partial bone loss
Pathology		Mature dense bone tissue	Confirmed ossification
Long-term follow-up (~5 yr)		No recurrence of symptoms	Complete resolution

3D = three-dimensional, CT = computed tomography.

## 3. Discussion

The cause of Eagle’s syndrome is poorly understood, but it is characterized by elongation of the SP and/or ossification of the SHL.^[20]^ Some authors hypothesize that genetic variations and endocrine dysfunctions in postmenopausal women are also causes of SHC ossification,^[20]^ and abnormal development of the SHC is also related to atlanto-occipital joint malformations.^[21]^ Morrison et al reported a case of familial ossification of the SHL, manifesting as autosomal dominant inheritance.^[22]^

Data from the literature indicate a wide variation in the frequency of SHC ossification, ranging from 1.4% to 83.6%.^[3,23–26]^ Research has revealed that long-term SHC ossification occurs in about 40.5% of patients, with both the number and length of ossifications tending to increase with age. The majority of these patients are asymptomatic, and only a small percentage (approximately 8%) of those with ossified SHC exhibit symptoms related to the condition,^[27]^ that is, Eagle’s syndrome. These findings suggest that the length of SHC ossification is not a major determinant of orofacial pain.^[24]^ When symptoms are present, there is no correlation between the severity of symptoms and the degree of ossification.^[24]^ As in our case, the patient had no history of trauma or tonsillectomy and had pain for only 4 months. This raises an interesting question: why do symptoms only appear recently when complete ossification of the SHC takes many years? Some authors hypothesize that an as-yet-undiscovered triggering mechanism has altered the soft tissue balance in the area, changing the muscle contraction pattern of the surrounding muscles, thereby causing symptoms.^[28]^ Some authors propose that it is due to the ossified structures stimulating surrounding tissues, including the carotid artery and cranial nerves VII, IX, and X, or due to the gradual loss of elasticity in the surrounding soft tissues over the years. In addition, these pathological conditions lead to reduced expansibility of the SHC, greatly increasing tension, affecting the ability of the hyoid bone to elevate, inhibit, and rotate with head and oral movements, causing compression and stimulation of surrounding neurovascular structures of the SHC, leading to pain.

The clinical presentation of Eagle’s syndrome is diverse, so its differential diagnosis is numerous, including temporomandibular joint disorder; trigeminal, sphenopalatine, glossopharyngeal, and superior laryngeal neuralgia; myofascial pain; mastoiditis; otitis media; giant cell arteritis; toothache (especially the third molar); tonsillitis; chronic pharyngitis, submandibular glanditis or sialolithiasis; esophageal diverticulum; benign or malignant tumors; true pharyngeal foreign body; migraine; cervical spondylosis; and neurosis.^[5,6,12–15]^ Clinically, when Eagle’s syndrome is suspected in a patient, palpation of the elongated SP or ossified SHL can further assist in diagnosis, and the final diagnosis can be confirmed by multilayer spiral CT 3D reconstruction.^[12–15]^ Multilayer spiral CT 3D reconstruction can provide clinicians with accurate data on the morphology, course, length, and anatomical relationship of the SP and surrounding tissues, accurately and stereoscopically displaying the SP itself and whether the SHL is ossified, accurately measuring the length of the SP and hyoid bone, providing key data and path selection basis for formulating surgical plans, and is therefore considered the gold standard for diagnosing SHC ossification.^[12–15]^ Langlais classified the radiographic appearance of ossification of the stylohyoid complex into 3 types: elongation, pseudoarthrosis, and segmentation, and according to the pattern of mineralization into 4 types: peripheral ossification, partial ossification, nodular, and complete ossification.^[23]^ Our case belongs to the pseudoarthrosis type and complete ossification type, with the right fully ossified SHC shown in the image having a pseudoarthrosis, dividing the ossified SHC into 2 parts, the upper segment about 6.5 cm long and the lower segment about 3.5 cm long, with ossification and thickening occurring at the junction of the SHL and the lesser horn of the hyoid bone.

The treatment methods for Eagle’s syndrome include conservative treatment and surgical treatment.^[14–17]^ The long-term efficacy of conservative treatment is not guaranteed, symptoms usually cannot be relieved or recur, and injection treatment is not completely noninvasive. Currently, shortening the ossified SHC through surgery is the most effective treatment for Eagle’s syndrome.^[14–17]^ There are 2 surgical approaches, oropharyngeal and extraoral.^[15–18]^ The oropharyngeal approach is suitable for patients with palpable SPs in the tonsillar fossa and chronic tonsillitis. Some literature reports that after bilateral SP shortening surgery through the oral approach, the patient developed severe trismus and moderate respiratory distress postoperatively.^[29]^ For patients who do not palpate the SP in the mouth, those with large SPs or those who fail the oral approach, especially patients with SHC ossification, should choose the extraoral approach.^[29]^ In the case reported in this article, the SHC was completely ossified and abnormally thick, so we chose the extraoral approach, with a clear surgical field, and completely excised the ossified SHC. Currently, there is no significant difference in efficacy between the oral and extraoral surgical approaches. Although some literature reports that the incidence of postoperative complications is higher with the oral approach compared to the extraoral approach,^[29]^ we believe that it is very important to choose the appropriate and less invasive surgical approach for different individuals.

## 4. Conclusion

We report a rare case of Eagle’s syndrome caused by complete ossification of the SHC accompanied by ossification of the lateral thyrohyoid ligament. As the clinical presentation of Eagle’s syndrome is diverse and lacks specificity, awareness and recognition of this rare condition are crucial for clinicians. Employing multilayer spiral CT with 3D reconstruction greatly enhances diagnostic accuracy and aids in the strategic planning of surgical interventions. Currently, surgery is the most dependable and effective treatment for Eagle’s syndrome, with the surgical approach being customized based on each patient’s unique condition.

## 5. Patient perspective

I had been suffering from persistent pain in my jaw and under my ear for months, and it was affecting my daily life and sleep. After trying several treatments at different hospitals without success, I was relieved to finally receive a clear diagnosis. The surgery resolved my pain completely, and I am very satisfied with the outcome and grateful to the medical team.

## Author contributions

**Formal analysis:** He Liu.

**Investigation:** Hui Li.

**Methodology:** Hui Li, Si-Qing Zhang, Lei Zhang, He Liu, Hai-Ying Zheng.

**Supervision:** He Liu, Hai-Ying Zheng.

**Writing – original draft:** Hui Li, He Liu.

**Writing – review & editing:** Si-Qing Zhang, Lei Zhang, Hai-Ying Zheng.
